# Effects of physical exercise interventions in frail older adults: a systematic review of randomized controlled trials

**DOI:** 10.1186/s12877-015-0155-4

**Published:** 2015-12-02

**Authors:** Carmen de Labra, Christyanne Guimaraes-Pinheiro, Ana Maseda, Trinidad Lorenzo, José C. Millán-Calenti

**Affiliations:** Research, Development and Innovation Department, Gerontological Complex La Milagrosa, Provincial Association of Pensioners and Retired People (UDP) from A Coruña, Avenida de Cádiz-5, E-15008 A Coruña, Spain; Gerontology Research Group, Department of Medicine, Faculty of Health Sciences, Universidade da Coruña, Campus de Oza, E-15071 A Coruña, Spain

**Keywords:** Exercise, Frail elderly, Physical activity, Functional capacity, Systematic review, Randomized controlled trial

## Abstract

**Background:**

Low physical activity has been shown to be one of the most common components of frailty, and interventions have been considered to prevent or reverse this syndrome. The purpose of this systematic review of randomized, controlled trials is to examine the exercise interventions to manage frailty in older people.

**Methods:**

The PubMed, Web of Science, and Cochrane Central Register of Controlled Trials databases were searched using specific keywords and Medical Subject Headings for randomized, controlled trials published during the period of 2003–2015, which enrolled frail older adults in an exercise intervention program. Studies where frailty had been defined were included in the review. A narrative synthesis approach was performed to examine the results. The Physiotherapy Evidence Database (PEDro scale) was used to assess the methodological quality of the selected studies.

**Results:**

Of 507 articles, nine papers met the inclusion criteria. Of these, six included multi-component exercise interventions (aerobic and resistance training not coexisting in the intervention), one included physical comprehensive training, and two included exercises based on strength training. All nine of these trials included a control group receiving no treatment, maintaining their habitual lifestyle or using a home-based low level exercise program. Five investigated the effects of exercise on falls, and among them, three found a positive impact of exercise interventions on this parameter. Six trials reported the effects of exercise training on several aspects of mobility, and among them, four showed enhancements in several measurements of this outcome. Three trials focused on the effects of exercise intervention on balance performance, and one demonstrated enhanced balance. Four trials investigated functional ability, and two showed positive results after the intervention. Seven trials investigated the effects of exercise intervention on muscle strength, and five of them reported increases; three trials investigated the effects of exercise training on body composition, finding improvements in this parameter in two of them; finally, one trial investigated the effects of exercise on frailty using Fried’s criteria and found an improvement in this measurement. Exercise interventions have demonstrated improvement in different outcome measurements in frail older adults, however, there were large differences between studies with regard to effect sizes.

**Conclusions:**

This systematic review suggested that frail older adults seemed to benefit from exercise interventions, although the optimal program remains unclear. More studies of this topic and with frail populations are needed to select the most favorable exercise program.

**Electronic supplementary material:**

The online version of this article (doi:10.1186/s12877-015-0155-4) contains supplementary material, which is available to authorized users.

## Background

According to the World Health Organization (WHO) [1], the proportion of people older than 60 is growing more rapidly than any other age group. Within this group, not everyone reaches old age successfully. Although the common rule is that increasing age is related to decreasing well-being and increasing levels of frailty [2, 3], it is also true that individuals with the same chronological age can vary in health and functional status [4].

The definition of frailty attempts to explain this discordance, and to do so, researchers can apply different approaches. One of them relates frailty to a physical phenotype describing only physical components [2]. According to this definition, the frailty syndrome is based on the presence of three or more of the following criteria: shrinking, weakness, poor endurance, energy slowness, and a low physical activity level. This frailty phenotype is the most extensively used assessment in different settings [5], and it classifies people into three main conditions: non-frail, pre-frail, and frail. Other definitions go beyond this one and encompass psychological components, such as having difficulties in activities of daily living (ADLs), for example, and psychological and social dimensions [6].

Frailty is a multidimensional concept that influences several domains, such as gait, mobility, balance, muscle strength, motor processing, cognition, nutrition, endurance and physical activity [7], and it is directly related to adverse consequences, such as falls, disability, the need for long-term care, hospitalization, and even mortality [2, 6, 8]. These adverse outcomes constitute a source of considerable healthcare expenditure, and it is known that the reduction of adverse outcomes could lead to an offset in medical costs [9]. In this sense, the clinical outcomes related to frailty should be treated to prevent the socioeconomic burden associated with this condition.

There is evidence suggesting that frailty is a potentially modifiable dynamic process characterized by frequent transitions between states over time. This definition suggests that specific interventions and health strategies could be used to prevent, postpone or even reverse the frailty phenomenon [10]. The American College of Sports Medicine’s (ACSM) [11] position states that participation in regular physical activity elicits a number of favorable responses that contribute to healthy aging. In this sense, physical exercise has demonstrated its beneficial effects in reducing the risk of many adverse outcomes, such as frailty [12], the number of falls [13, 14], poor mental health [15, 16], decreased cognitive function [17], decreased cardiac and pulmonary function [18–20], decreased physical function, such as balance [13, 21, 22], gait [21] and mobility [23], and poor muscular power and functional capacity [13, 24]. All of this accumulated evidence indicates that physical exercise, either in its aerobic or strength form, is fully recommended both in healthy older adults and in elderly people with chronic diseases and disabilities [11].

Although numerous interventions have been developed to improve the outcomes of frail elderly people, a major obstacle found by researchers to success in such interventions has been the difficulty of comparing the studies retrieved, due to the differences in the diagnosis of frailty. Considering this difficulty and assuming that exercise training produces beneficial effects in the elderly, the major goal of this systematic review of randomized, controlled trials (RCTs) was to investigate the benefits of exercise programs in frail elderly people, considering only those studies where frailty had been defined.

## Methods

### Data sources and search strategy

An extensive literature search of electronic databases, including PubMed, Web of Science and the Cochrane Central Register of Controlled Trials, was performed in three phases. In the first phase, without limiting the years of the search and introducing the search terms “aging”, “frailty”, “frail elderly”, “aerobic exercise” and “strength exercise”, we extracted the theoretical framework that is used as the basis for the current review. In the second phase (started in January 2013), the search was restricted to the last 10 years (January 2003–December 2012). Later, in the third phase, an updating of the literature (from January 2013 to June 2015) was performed. In the second and third phase, to select RCTs in which the intervention was performed with aerobic or resistance training in frail older people, we searched articles using the following keywords and Medical Subject Headings: [(randomized controlled trial) AND (frail elderly) AND (aerobic exercise) OR (randomized controlled trial) AND (frail elderly) AND (strength OR resistance exercise)].

### Inclusion criteria and quality assessment

Based on the titles, abstracts and some parts of the articles when needed, we screened the literature to select those articles meeting the inclusion criteria. We described all of the studies that met the inclusion criteria according to the following:

- Subjects: Frail elderly people, defined with a clear operational definition/measurement of frailty;
- Study design: Original analysis of an RCT citing some type of aerobic or resistance training intervention for the intervention group(s) (IGs), and the control group (CTL) receiving no treatment, maintaining its habitual lifestyle or a home-based low level exercise program;
- Outcomes: Studies examining, as a primary outcome, the effects of the intervention on the domains of frailty and/or physical capacity and/or functional capacity of the sample; and
- Language: Only publications in English were considered.
- Exclusion criteria: Duplicated studies, non RCTs, studies in which both aerobic and resistance training coexisted in the intervention, secondary analysis of data sets, abstracts, reviews, descriptive studies and studies based on the description of a protocol, and studies based on the point of view of the authors were excluded. However, those studies in which aerobic training was included in the warm up or cool down and interventions based on resistance training, and vice versa, were included for review.

Data extraction was standardized according to the following terms: (i) design; (ii) objective; (iii) sample characteristics; (iv) type, intensity, frequency, and duration of the intervention; (v) measurement tools; and (vi) findings. Because of the heterogeneity of the study designs, a narrative synthesis approach was performed to examine the results, rather than a meta-analysis. Cohen’s *d* values were reported as indicators of effect size (ES). We interpreted the importance of the ES using the benchmarks for “small ES” (*d* = 0.2), “medium ES” (*d* = 0.5) and “large ES” (*d* = 0.8) offered by Cohen (1988) [25].

The methodological quality of each RCT was rated using the Physiotherapy Evidence Database (PEDro) scale [26]. The PEDro scale is an instrument for the methodological quality assessment of RCTs in physical therapy and exercise studies. The items on the PEDro scale were derived from a Delphi consensus procedure [27], and there are 11 items (see Table 1). The PEDro scale scores can range from 0 to 10, with a higher score indicating better methodological quality. Responses to items 2 to 11 are summed to create a total score, and item 1 relates to external validity. The reliability of this scale was evaluated with acceptable good results in intraclass correlation coefficients 0.56–0.91 [26, 28]. Judgment by two independent raters was compared and, when necessary, discussed.

**Table 1:** PEDro scale rating [26]

Reference	Eligibility criteria	Random allocation	Concealed allocation	Group similar at baseline	Blinded subjects	Blinded therapist	Blinded assessors	Less than 15 % dropouts	Intention-to-treat analysis	Between-group comparisons	Point measure and variability	PEDro score
Binder 2005 [38]	1	1	0	1	0	0	0	1	1	1	1	6
Cadore 2014 [13]	1	1	1	0	0	0	1	1	1	1	1	7
Faber 2006 [32]	1	1	1	1	0	0	1	1	1	1	1	8
Fairhall 2014 [33]	1	1	1	1	0	0	0	1	1	1	1	7
Giné-Garriga 2010 [34]	1	1	1	1	0	0	1	1	1	1	1	8
Giné-Garriga 2013 [35]	1	1	1	1	0	0	1	1	1	1	1	8
Kim 2015 [37]	1	1	1	1	0	0	1	1	1	1	1	8
Latham 2003 [36]	1	1	1	1	0	0	1	1	1	1	1	8
Lustosa 2011 [31]	0	1	0	0	0	0	1	0	1	1	0	4
Total	8	9	7	7	0	0	7	8	9	9	8	7.1

Ethical approval was noted for all of the published papers included in this review.

## Results

### Study selection

**Fig. 1:**
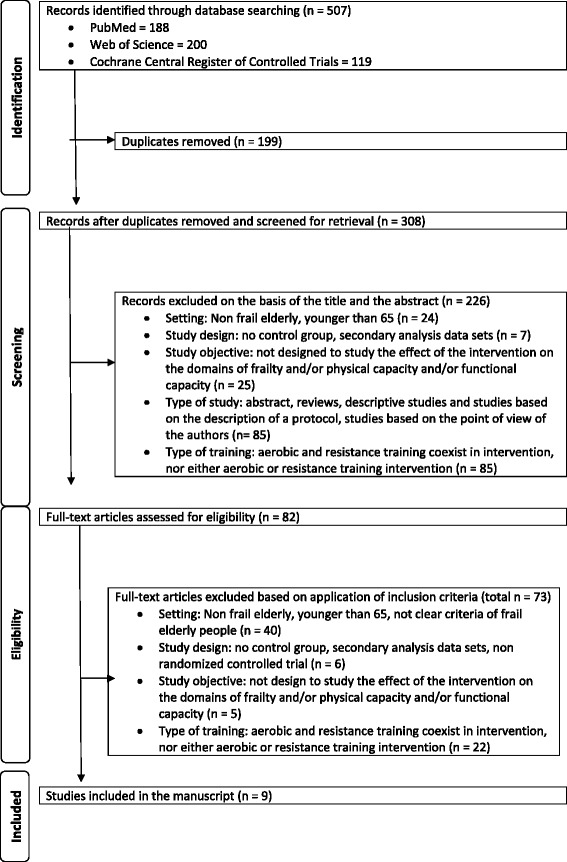
Flow diagram of study selection

Figure 1 summarizes the results of the different steps to identify appropriate articles for the review. The Preferred Reporting Items for Systematic Reviews and Meta-Analyses (PRISMA) Statement was followed [29, 30] (see Additional file 1). The database search identified 507 articles, and after duplicate removal, 308 were considered potentially relevant and were screened for relevant content. From them, 226 were excluded on the basis of the title and the abstract, and 82 were retrieved for full-text assessment of eligibility. In the next phase, 73 of the 82 full-text articles were excluded based on the inclusion criteria. Forty trials were excluded for not meeting the setting characteristics: six for not meeting the study design, five for not meeting the study objective, and 22 for not meeting the type of training characteristics. Finally, nine studies met the inclusion criteria and were included in this review.

### Methodological quality

PEDro scores ranged from 4 to 8 points, with a mean score of 7.1 (Table 1). All of the selected studies but one [31] scored 6 or more, indicating the high quality of the selected trials. All of the studies except for one [31] specified the eligibility criteria. In all of the studies, the subjects were randomly allocated to groups, and seven of them had concealment of allocation [13, 32–37]. Seven studies showed the similarities at baseline [32–38]. None of the trials had blinded participants or therapists, and seven had blinded assessors [13, 31, 32, 34–37]. Eight trials had retention rates of 85 % or greater [13, 32–38], and all of the studies met the intention-to-treat analysis criteria. All of the studies applied statistical analysis to group differences, and eight of them reported point estimates and measurements of variability [13, 32–38]. No studies were excluded on the basis of their methodological quality (see Table 1).

### Participants and study characteristics

The included articles encompassed a sample of 1067 older people (71.8 % women), with a mean age of 82.5 ± 4.3 years old. A total of 562 participants were community-dwelling elders, 262 lived in residential care facilities, and 243 were recruited from an acute care and rehabilitation teaching hospital. The identification of frail older people was based on Fried’s criteria [13, 31, 33, 37], on indicators adapted from Fried’s criteria [32], on Winograd’s frailty scale [36], on a modified Physical Performance Test score, reporting the difficulty and/or assistance with up to two instrumental activities of daily living and/or one basic activity of daily living, and a peak aerobic power between 10 and 18 mL kg^−1^ min^−1^ [38], on two tests of physical abilities, and according to two questions from the Center for Epidemiological Studies depression scale [34, 35].

Four studies were conducted in Europe [13, 32, 34, 35], two in Australia/Oceania [33, 36], one in the United States of America [38], one in Brazil [31], and the last one in Asia [37]. While four studies did not have follow-up measurements after the intervention [13, 31, 33, 38], five studies recorded follow-up measurements ranging from 4 to 12 months [32, 34–37]. With regard to the type of intervention, the core of the selected articles was centered on resistance training exercises [13, 31–38] (Table 2).

**Table 2:** Characteristics of the included studies

Reference	Sample	Setting	Identification of frailty	Intervention characteristics	Outcome measures	Findings (Cohen’s *d*)
Binder 2005 [38]	*n* = 91 from the USA Age, mean ± SD: 83.0 ± 4.0 54 % women	Community dwelling	Screening instruments and procedures [12]: Modified Physical Performance Test score between 18 and 32 (maximum 36); reporting the difficulty and/or assistance with up to two instrumental and/or one basic ADL; and a peak aerobic power between 10 and 18 mL kg^-1^ min^-1^	Multi-component PRT 9 months 3/week 60-90 min/session Initial goal: 1-2 sets 6-8 repetitions 65% 1-RM Final goal: 3 sets 8-12 repetitions 40-60% 1-RM 1-RM strength in six different exercises (knee extension, knee flexion, seated bench press, seated row, leg press, biceps curl), performed bilaterally in a weightlifting machine Upper and lower extremities	Strength: skeletal muscle strength (maximal voluntary muscle strength for knee extension and flexion) Body composition: Total body DEXA	Significant increase in knee extension with the intervention (*d* = 0.62) Knee flexion strength showed no effect with the intervention Intervention induced greater increases in total (*d* = 0.20) and regional (*d* = 0.19) FFM but no changes in fat mass
Cadore 2014 [13]	*n* = 24 from Europe Age, mean ± SD: 91.9 ± 4.1 70 % women	Institutionalized	Fried’s criteria [2]	Multi-component PRT 12 weeks 2/week 40 min/session 8-10 repetitions 40-60% 1-RM Two exercises for the leg extensor muscles (bilateral leg extension and bilateral knee extension muscles) and one exercise for upper limbs (seated bench press), performed on a resistance variable exercise machine Upper and lower extremities	Falls: Incidence Mobility: 5 meter walking tests at their habitual speed; TUG; chair rising ability (the most rises in 30 sec) Balance: FICSIT-4 Functional disability: ADLs using BI Strength: Isometric upper and lower limb muscle strength Body composition: fat muscle infiltration	Exercise training significantly reduced the incidence of falls (*d* = 2.71) Walking ability did not change with the intervention Exercise training significantly improved the time spent on the TUG (*d* = 0.42) Significant change in the chair-rising ability test in the intervention group (*d* = 0.89) Exercise training improved balance (*d* = 0.72) Exercise training improved functional ability (*d* = 1.17) Significant increase in knee extension with the intervention (*d* = 1.74) Upper body muscle strength did not significantly change with the intervention Intervention induced a decrease in fat muscle infiltration (Quadriceps femoris, *d* = 0.20; and knee flexor, *d* = 0.10)
Faber 2006 [32]	*n* = 238 from Europe Age, mean ± SD: 84.9 ± 6.0 79 % women	Institutionalized	Frailty indicators adapted from Fried’s criteria [2]	Multi-component functional walking and in balance exercises 20 weeks 1/week for 4 weeks 2/week for 16 weeks 90 min/session Exercise without machines focused on balance, mobility, and transfer training. They included standing up from a chair, reaching and stepping forward and sideward, heel and toe stands, walking and turning, stepping on and over an obstacle, staircase walking, tandem foot standing, and single-limb standing Lower extremities Tailored to the needs of each participant	Falls: incidence Mobility: POMA; walking tests: 6 meters as fast as possible; TUG; chair rising ability (the time needed to stand up and sit down 5 consecutive times as fast as possible) Balance: POMA; FICSIT-4 Functional disability: ADL and instrumental ADL using GARS	Exercise training significantly reduced the incidence of falls in the pre-frail elderly sample. In the frail subgroup, the risk of becoming a faller increased with the intervention Positive effect of the intervention on mobility in the pre-frail subgroup. In the frail subgroup, mobility worsened after the intervention Small, but significant, positive intervention effect in POMA score in the exercise group, compared with the control group Exercise training showed no effect on functional ability
Fairhall 2014 [33]	*n* = 241 from Australia/Oceania Age, mean ± SD: 83.3 ± 5.9 68 % women	Community dwelling	Fried’s criteria [2]	Multi-component exercise intervention: Home program of balance and lower limb training based on the WEBB program 12 months 3-5/week 20-30 min/session Exercises without machines Lower extremities Tailored to the needs of each participant	Falls: incidence; risk of falls (Physiological Profile Assessment [PPA]; short physical performance battery [SPPB]); 4-m walking tests Strength: Lower body strength (knee-extension strength as a component of the PPA)	Exercise training did not significantly reduce the incidence of falls Exercise training found a better postural sway (*d* = 0.09) Significant increase in leg muscle extension with the intervention (*d* = 0.03) Significant improvements in mobility (SPPB score, *d* = 0.40; and gait speed, *d* = 0.20)
Giné-Garriga 2010 [34]	*n* = 51 from Europe Age, mean ± SD: 84.0 ± 2.9 61 % women	Community dwelling	Two tests of physical abilities [51, 52] and according to two questions from the Center for Epidemiological Studies depression scale [2]	Multi-component functional based circuit training 12 weeks 2/week 45 min/session 1-2 sets 6-8 repetitions 1 day of balance-based activities and 1 day of lower-body strength-based exercises, combined with function-focused activities. Exercises without machines Lower extremity exercises included activities such as rising from a chair, stair climbing, knee bends, floor transfer, lunges, leg squats, leg extension, leg flexion, calf raises, and abdominal curls using ankle weights	Mobility: walking tests: 8 meters at their habitual speed and as fast as comfortably possible; MTUG (modified TUG test) Functional disability: ADL using BI Strength: lower body strength (knee-extension and flexion strength)	Walking ability improved with the intervention (Balance measures: semitandem *d* = 4.65, tandem *d* = 6.62, and single leg *d* = 7.78; Gait speed measures: normal *d* = 3.50 and fast *d* = 3.50) Exercise training significantly improved the time spent on the MTUG (assessment questionnaire *d* = 8.24, and total time *d* = 4.61) Exercise training improved functional ability (BI score *d* = 1.08) Significant increase in leg muscle extension with the intervention *d* = 3.50)
Giné-Garriga 2013 [35]	*n* = 51 from Europe Age, mean ± SD: 84.0 ± 2.9 61 % women	Community dwelling	Two tests of physical abilities [51] and according to two questions from the Center for Epidemiological Studies depression scale [2]	Multi-component functionally based circuit training 12 weeks 2/week 45 min/session 1-2 sets 6-8 repetitions 1 day of balance-based activities and 1 day of lower-body strength-based exercises, combined with function-focused activities. Exercises without machines Lower extremity exercises included activities, such as rising from a chair, stair climbing, knee bends, floor transfer, lunges, leg squat, leg extension, leg flexion, calf raise, and abdominal curl using ankle weights	Falls: fear of falling (Activities-specific Balance Confidence [ABC] scale)	Exercise training improved the fear of falling (*d* = 1.10)
Kim 2015 [37]	*N* = 131 from Asia Age, mean ± SD: 80.7 ± 2.8 100 % women	Community dwelling	Fried’s criteria [2]	Physical comprehensive training 12 weeks 2/week 60 min/session 30 minutes of strengthening exercises plus 20 minutes of balance and gait training Strength exercises performed in progressive sequence from the seated to standing positions, and progressive resistance was applied through Thera-bands, with increasing repetition with each exercise Lower extremities consisted of leg extensions, hip flexions, and more. Upper body exercises included double-arm pull downs, bicep curls, and others	Mobility: walking speed; TUG Strength: Grip strength and isometric knee extension strength Body composition: Total body DEXA Frailty status	Walking speed did not change with the intervention Exercise training improved the time spent on the TUG (*d* = 0.64) No increase in knee extension with the intervention Upper body muscle strength did not significantly change with the intervention No effect on body composition of the intervention Exercise training and exercise training plus nutrition supplementation significantly improved frailty status
Latham 2003 [36]	*n* = 243 from Australia/Oceania Age, mean ± SD: 79.1 ± 6.9 53 % women	Teaching hospitals	Winograd’s frailty scale [53]	Home-based resistance training 20 weeks 3/week Initial goal: 3 sets 8 repetitions 30-40% 1RM for 2 weeks Final goal: 3 sets 8 repetitions 60-80% 1RM Accomplished goal: 3 sets 8 repetitions 51% 1RM ±13% Adjustable ankle cuff weights Lower extremities	Falls: incidence; fear of falling Mobility: 4 meter walking tests; TUG Balance: BBS Functional disability: ADL (BI) and participation in higher non-ADL levels of activity (Adelaide Activity Profiles) Strength: maximal isometric knee extensor strength	Exercise training did not significantly reduce the incidence of falls Walking ability did not change with the intervention Exercise training did not change TUG measurements Balance was not affected by the intervention No intervention effect on ADL with exercise training No effect on leg muscle extension with the intervention
Lustosa 2011 [31]	*n* = 48 from Brazil Age, mean ± SD: 72.0 ± 4.0 100 % women	Community dwelling	Fried’s criteria [2]	Body weight resistance training 10 weeks 3/week 60 min/session 3 sets 8 repetitions 70% 1RM Ankle weights with loads ranging from 0.5 to 3 kg Lower extremities	Mobility: 10 meter walking tests at their habitual speed; TUG Strength: Muscle strength of knee extensor	Walking ability improved with the intervention (*d* = 0.69) Exercise training significantly improved the time spent on the TUG (*d* = 0.17) Significant increase in leg muscle extension with the intervention (*d* = 0.05)

*d* = Cohen’s *d* (effect size). A value of 0.2 indicates a small effect, 0.5 a medium effect and 0.8 a large effect [25]. *PRT* progressive resistance exercise training, *1-RM* one-repetition maximum, *WEBB* weight-bearing for better balance program, *DEXA* body dual energy x-ray absorptiometry *FFM* fat-free mass, *FICSIT-4* frailty and injuries: cooperative studies of intervention techniques–4 static balance tests, *BI* Barthel index, *ADL* activities of daily living, *POMA* performance oriented mobility assessment, *TUG* time up-and-go test, *GARS* Groningen activity restriction scale, *PPA* physiological profile assessment, *SPPB* short physical performance battery, *MTUG* modified TUG test, *ABC* activities-specific balance confidence scale, *BBS* Berg balance scale, *FFM* fat-free mass

### Intervention characteristics

The intervention characteristics are summarized in Table 2. Six studies included multi component exercise interventions [13, 32–35, 38]. In two of these six studies, the intervention program involved progressive resistance exercise training (PRT) [13, 38]: one used an intervention home program of balance and strength, based on the Weight-bearing for Better Balance (WEBB) program [33]; one investigated two exercise programs, functional walking, consisting of exercises related to daily mobility activities, and balance exercises, consisting of exercises derived from principles of Tai Chi [32]; and two applied a functional-based circuit training (FCT) program, being a combination of functional balance and lower-body strength exercises [34, 35]. The remaining three trials used non-multi-component programs, based on strength interventions [31, 36, 37].

The intervention lasted less than 6 months in seven of the trials [13, 31, 32, 34–37], 9 months in another trial [38], and 1 year in the last one [33]. The frequency of the training programs ranged from 2 to 3 times per week in almost all of the trials, except for one, which ranged from 3 to 5 times per week [33]. All but one study [36] reported the length of each session, ranging from 20 to 30 min [33] to 60–90 min [32, 37, 38]. Eight [13, 31, 32, 34–38] of the nine [33] selected articles provided a detailed description of the program with regard to the intensity of the exercise intervention. Two of the trials used a weightlifting machine [13, 38]. In one of them, the participants started with an initial configuration of 1–2 sets of 6–8 repetitions of each exercise at 65 % of their one-repetition maximum (1-RM) with the goal of three sets of 8–12 repetitions of each exercise at 40–60 % 1-RM [38], and in the other, the participants underwent a configuration of 8–10 repetitions of each exercise at 40–60 % 1-RM [13]. Two other trials used functional strength exercises without the help of any machine [34, 35]. Both of them encompassed exercises such as rising from a chair, stair climbing, knee bends, floor transfer, lunges, leg squats, leg extension, leg flexion, calf raises, and abdominal curls using ankle weights. The configuration of the exercises was 1–2 sets of 6–8 repetitions for each exercise. Two of the trials employed ankle weights as a core of the training program [31, 36]. In one of them, the participants underwent a configuration of three sets of eight repetitions of each exercise with a final goal set at 60–80 % of their 1-RM (initial goal set at 30–40 % 1-RM, accomplished goal 51 ± 13 % 1-RM) [36], and in the other, the participants underwent a configuration of three sets of eight repetitions of each exercise at 70 % 1-RM with loads ranging from 0.5 to 3 kg [31]. One of the trials used Thera-bands as a nucleus of the training program [37]. Lower and upper body exercises consisted of leg extensions, hip flexions, double-arm pull downs, bicep curls, and others. In this trial, milk fat globule membrane (MFGM) supplementation was also used to study its combined and separate effects with exercise training. Finally, one of the trials was designed to prevent falls with two programs: a functional walking program consisting of ten exercises focusing on balance, mobility, and transfer training, such as standing up from a chair, reaching and stepping forward and sideward, heel and toe stands, walking and turning, stepping on and over an obstacle, staircase walking, tandem foot standing, and single-limb standing; and an in balance program inspired in Tai-chi [32].

The intervention was targeted to the upper and lower extremities in three of the studies [13, 37, 38] and to the lower extremities in the remaining six [31–36]. In only two of the trials, the intervention was tailored to the needs of the participants [32, 33].

### Outcome measurements

#### Falls

Falls were examined in five studies [13, 32, 33, 35, 36] by measuring the incidence of falls [13, 32, 33, 36], risk of falls [33], and fear of falling [35]. The effects of exercises on the incidence of falls were controversial among the studies. In two of them [13, 32], falls were significantly reduced after exercise training, at least in the pre-frail elderly sample [32], but not in the frail subgroup, in which the risk of becoming a faller increased with the intervention [32]. In two other trials, no significant effects of the training were found in the incidence of falls [33, 36]. The only study considering the risk of falls found better effects on risk factors for falls with the intervention [33]. Finally, the fear of falling also improved after the intervention [35].

#### Mobility

Several aspects of mobility were measured in six of the nine trials [13, 31, 32, 34, 36, 37]. One study used four combined standardized physical performance tests to develop a single measurement of mobility [32]. The authors found different results depending on the sample subgroup. In the frail subgroup, the physical performance score was lower after the intervention, whereas there was a positive effect of the intervention on the physical performance score in the pre-frail subgroup. The other five trials used individualized standard measurements of mobility [13, 31, 34, 36, 37]. All of them measured walking ability with several variations of walking tests, ranging from walking 4 to 10 m at habitual speed or as rapidly as comfortably possible. While in two of the trials, walking ability improved with the intervention [31, 34], in the other three trials, there were no changes in the IG [13, 36, 37], with one of these trials finding a significant decrease in the gait velocity of the CTL [13]. Mobility using the Timed up-and-go test (TUG) was measured in five trials [13, 31, 34, 36, 37]. While in four of them, a significant improvement was found in the time spent on the TUG test in the IG compared to the CTL [13, 31, 34, 37], one study found no effect of the intervention on this measurement [36]. Chair-rising ability was measured in one of the four studies using individualized standard measurements of mobility [13], finding a significant increase in performance on this test in the IG and no changes in the CTL. One study also employed the Performance Oriented Mobility Assessment (POMA) as a measurement of mobility [36]. It found a small, but significant, positive effect of the intervention on the POMA score in the IG, compared with the CTL.

#### Balance

Balance was measured in three of the selected trials [13, 32, 36]. The first one used the Frailty and Injuries: Cooperative Studies of Intervention Techniques–4 (FICSIT-4) static balance tests [13], the second trial used the FICSIT-4 tests of static balance, but the result was reported as a combined measurement of four standardized physical performance tests [32] (the result of this combined measurement was discussed in the mobility section), and the last one used the Berg Balance Scale (BBS) [36]. After training exercises, balance improved in one of the trials [13], and in the others, balance was not affected by the exercise intervention [36].

#### Functional ability

Functional ability was measured in four of the nine trials [13, 32, 34, 36]. Three used the Barthel Index (BI) [13, 34, 36] (one of them also used the Adelaide Activity Profiles [36]), and the other used the Groningen Activity Restriction Scale (GARS) [32]. Interventions showed positive results in improving functional ability in two of the trials [13, 34], whereas in the other two trials, no effects of the intervention were found in this measurement [32, 36].

#### Muscle strength

Seven of the nine trials measured muscle strength [13, 31, 33, 34, 36–38]. Lower limb strength was measured in all of them, measuring knee extension. Knee flexion was measured in two of the trials [34, 38], and hip flexion was measured in one trial [13]. Upper muscle strength was measured in two trials only [13, 37]. One of the trials measured the grip strength in the right hand [13], and the other trial measured grip strength in the dominant hand. Five of the seven trials measuring knee extension showed a significant increase in this measurement with the intervention [13, 31, 33, 34, 38]. Only two trials showed no effect of the intervention on this measurement [36, 37]. None of the trials measuring flexion strength [34, 38] found any improvement with the intervention. Similarly, the two trials measuring upper muscle strength found no significant changes with the intervention [13, 37].

#### Body composition

Two studies examined body composition [37, 38], measuring it with total body dual energy x-ray absorptiometry (DEXA). While one of these studies [38] found that low to moderate intensity PRT induced greater increases in total and regional fat-free mass (FFM) and isokinetic muscle strength but no changes in fat mass, compared to a home-based, low intensity exercise program, the other study [37] did not have any effect on this parameter. Another study [13] examined muscle tissue attenuation (indicative of fat infiltration) using computed tomography scans at the midthigh of the left quadriceps femoris using a 64-row CT scanner. The results of this study revealed that a multi-component exercise program including muscle power training induced a decrease in muscle fat infiltration.

#### Frailty status

One study examined frailty status [37], which was defined as the presence of three or more of the five frailty criteria [2]. Reversal of frailty was defined as the percentage of subjects who were defined as frail at baseline but who decreased to two or below at post-intervention. This study found that 3 months of exercise had an effect on frailty status, reversing the frailty status in comparison to the CTL group. However, when the 3-month exercise was combined with the nutrition supplementation program (MFGM), the effect on frailty reversal was maximized.

## Discussion

This systematic review, which aimed to retrieve recent evidence examining the potential role of physical exercise interventions in frail older adults, provided evidence that physical exercise has positive effects on most of the outcome measurements included in this current revision. There were, however, large differences between studies with regard to the effect sizes. Except for one trial [36], consistent findings were found regarding the effects of the evaluated exercise programs on frailty and/or physical capacity and/or functional capacity. To perform this review, we used strict criteria to define frailty in older people, indicating that trials including the word “frail” in the title or in the abstract, if not rigorously defined, were not included. Due to the lack of consensus about measurements for detecting frailty, the uniformity of the target groups might have been reduced; however, all of the trials were considered to have sufficient methodological quality to be included in this review.

Two of the trials applied single lower extremity strength training [31, 36]. While one of them showed significant improvements in mobility and strength (although with a trivial practical significance) [31], the other one did not find any effects on falls, mobility, balance, functional ability or strength [36]. These apparently negative results could be explained because of the limited duration of the program (only 10 weeks) or because the exercise program addressed one muscle group (quadriceps), thus limiting greater improvements in performance. The contradicting results observed in the trials using only strength training of the lower extremities could be sorted out by including strength training as part of a more complete program, such as a multi-component exercise program. In this review, the trials with multi-component physical exercise programs focused on resistance and/or balance and/or flexibility exercises [13, 33–35, 38] (the exercise program of Faber et al. [32] did not include strength training as part of the multi-component exercise program). All of the trials reported statistically significant effects for falls, mobility, balance, functional ability, muscle strength and body composition, except for one trial that found no changes in walking ability with the intervention [13] and another trial in which exercise training did not significantly reduce the incidence of falls [33]. Although most of the literature in this field has supported that multi-component exercises appear to be the most effective interventions to improve the overall health status of frail elderly people [13, 39], our search strategy focused on studies with a variety of exercise programs and with a broad range of methods to evaluate the results. Because of this heterogeneity, it is difficult to draw a conclusion about the single best program to apply for a specific frail population. It could be suggested that programs targeting more than one physical component (strength, endurance, balance, flexibility) promote better performance with regard to the global functional capacity of older adults. Moreover, the variety of interventions in the CTL (regular care, low intensity exercises, standard exercises for activities of daily living, etc.), although improving the accuracy and generalization of external data, could also influence the specific effects of training, compared with other factors. Consequently, this variety could act as a confounder to determine the best intervention program.

The literature has shown that participation in regular physical activity protects against diverse components of frailty syndrome [40], improving the quality of life of older people [35] by increasing balance and mobility and reducing falls, as well as institutionalization, hospitalization and mortality. Not all of the studies analyzed in this review included interventions only addressing muscle strengthening, but they also combined programs addressing activities of daily living, walking, balance, nutrition supplementation, and others, potentially increasing the beneficial effects of the physical activity aiming to improve frailty. All of the reviewed studies apart from one [36] showed an increase in muscle strength and muscle extension, along with improvements in mobility, balance, functional capacity, and frailty status, suggesting positive effects on frailty syndrome. However, the clinical significance of some results is low or very low, with small to very small effect sizes [31, 33].

A critical component of resistance training programs is the intensity or the amount of weight being lifted. Four of the studies offered a detailed explanation of their programs [13, 31, 36, 38], with the majority of the studies using three sets of eight 1-RM at 40–80 %, with rest intervals. Research has shown that intensities ranging from 70 to 95 % of 1-RM significantly increase muscle strength [41]. In the trial of Latham et al. [36] and as a result of complaints from participants about muscle soreness, the therapists decreased the intensity of the weights to 30–40 % of 1-RM during the first 2 weeks of the program. This change could theoretically have moved the program away from the primary goal of strength training and could be the reason that it did not find significant differences between the CTL and the IG. In any case, this result suggested that, despite frailty conditions in the elderly, they are able to participate in a personalized strength training program that considers their own limits. This fact is very important because the exercises generally recommended to the elderly population used to be intense and tiring.

Obesity is a condition that exacerbates with age, producing a decline in the physical function of older adults and causing frailty and a reduction in the quality of life [42, 43]. Numerous studies have consistently shown that exercise benefits the body composition of healthy older adults [44]. Two of the nine trials included in this review studied the effects of PRT on the body composition of frail elderly people [13, 38], finding that the intervention induced greater increases in total and regional FFM and a decrease in the fat muscle infiltration, but no changes in fat mass. However, these results showed a small effect size. Moreover, one of the trials studied strengthening exercises plus balance and gait training in body composition [37], finding no significant effects on this measurement.

One of the nine studies included in this review had the specific goal of studying the reversal status in Fried’s frailty criteria [37]. This trial, which specifically investigated the combined and separate effects of exercise and MFGM supplementation on frailty, found that exercise training improved frailty status. However, the combined effect of exercise plus nutritional supplementation is perhaps the most beneficial option to improve frailty in elderly women. Although there has been increased interest regarding the value of nutritional supplementation for physical parameters [45], the vast majority of articles are performed in young adults. The trial of Kim et al. (2015) [37] confirmed the added value of nutritional supplementation on exercise interventions in the elderly. For the optimal effects of exercise, it is important that older people adhere to the prescribed exercise program [46]. Although we did not study the adherence of elderly people to the prescribed exercise programs, most of the studies included in this review reported small withdrawals from the exercise programs (lack of adherence) [13, 33–36, 38]. This fact could be mainly due to the typology of the participants -- frail elderly people --- or because adherence to programs is known to decrease in older people when more exercises are added to the program [47].

Although there have been several systematic reviews published addressing the issue of the benefits of exercise in frail elderly people [48–50], a decisive conclusion about the optimal program to improve the global capacity of frail elders remains unclear. Because of that, and because the topic of frailty is characterized by a high level of scientific publications, updatings to this literature are mandatory. With this systematic review, we provided an update of the literature. Of the three systematic reviews previously mentioned, two performed meta-analyses [49, 50]. Chou et al. [49] aimed with a meta-analysis to determine the effects of exercise training on the physical functions, performance of ADL, and quality of life of frail elderly people. Similar to our conclusions, they determined that exercise was beneficial in improving physical function and performance of ADLs. The meta-analysis of De Vries et al. [50], aimed to assess the effects of physical exercise interventions on mobility, physical functioning, physical activity and quality of life in elderly people with mobility problems, disability and/or multi-morbidity. Similar to us, but also including nonfrail and prefrail elders, they concluded that physical exercise interventions improved mobility and physical functioning. The systematic review of Cadore et al. [48] studied the effects of exercise programs on muscle strength, balance, gait ability, and the risk of falls, finding that multi-component interventions seemed to be the best strategy for improving those outcomes in frail elderly people.

Although this review presented well-defined inclusion criteria, we did find several methodological limitations. Considering the characteristics of the interventions and the outcome measurements, we observed great variety among the different studies. The most recommended intervention in frail subjects was multi-component training, which offers a broad variety of exercises with an enormous number of possibilities and combinations. Regarding the outcome measurements of the different trials, we should say they are the most commonly used measurements to assess the benefits of physical activity in frail elderly people. Additional limitations of this review were the few studies performed in institutionalized and hospital settings to perform significant comparisons among them and the small sample sizes of some of the trials, not only making the number of subjects in the IG and the GTL small, but also limiting the strength of the conclusions. Finally, another limitation of the current review was the lack of consensus in the definition of frailty and the use of various different criteria to define frailty, which reduced the uniformity of the target groups.

Although this systematic review provided evidence that physical exercise has positive effects on frail older adults, we should consider that there was great variety among the studies concerning sample size, degree of frailty, types of interventions and types of assessments. Besides, some findings should be interpreted cautiously because power analysis showed very small or small effect sizes. Moreover, the absence of changes in some of the outcomes explored in this analysis could indicate that exercise prescriptions must be carefully adapted to the sample of frail older people. A strong point of this analysis is that it is focused on a population of frail elderly people, defined with a clear theoretical or operational definition of frailty.

## Conclusions

This systematic review showed that exercise training addressed to frail elderly people could improve several aspects of their physical function. Although we suggested that multi-component exercise programs including some type of resistance training would promote better performance in the global functional capacity of frail older adults, the optimal program remains unclear. More studies regarding this topic and with this specific population are needed to determine the most favorable exercise program.

## Additional file

Additional file 1:
**PRISMA 2009 Checklist.** (DOC 62 kb)

## Abbreviations

1-RM: One-Repetition Maximum
ABC: activities-specific balance confidence scale
ACSM: American College of Sports Medicine
ADL: activities of daily living
BBS: Berg Balance Scale
BI: Barthel Index
CTL: control group
DEXA: body dual energy x-ray absorptiometry
FCT: functional-based circuit training program
FFM: fat-free mass
FICSIT-4: Frailty and Injuries: Cooperative Studies of Intervention Techniques–4 (FICSIT-4) static balance tests
GARS: Groningen Activity Restriction Scale
IG: intervention group
MTUG: modified timed up-and-go test
PEDro: physiotherapy evidence database scale
POMA: Performance Oriented Mobility Assessment
PPA: physiological profile assessment
PRT: progressive resistance exercise training
RCT: randomized and controlled clinical trials
SPPB: short physical performance battery
TUG: time up-and-go test
WEBB: weight-bearing for better balance program
